# Neuropsychopharmacology in the era of artificial intelligence and biomolecule prediction software

**DOI:** 10.1038/s44277-025-00038-9

**Published:** 2025-06-30

**Authors:** Rubén A. García-Reyes, Laura N. Massó Quiñones, Hajin Ruy, Daniel C. Castro

**Affiliations:** 1 Biophotonics Research Center, Mallinckrodt Institute of Radiology, Washington University School of Medicine in St. Louis, St. Louis, MO 63110 USA; 2 Roy and Diana Vagelos Division of Biology and Biomedical Sciences, Washington University School of Medicine in St. Louis, St. Louis, MO 63110 USA; 3 College of Arts and Sciences, Washington University in St. Louis, St. Louis, MO 63130 USA

**Keywords:** Neuroscience, Computational neuroscience

## Abstract

The development and adoption of artificial intelligence (AI) provides moonshot opportunities to redefine how we generate treatments for neuropsychiatric disease. Despite the rapid advancement of AI across biomedical spheres, its implementation in drug discovery, proteomics, and neurobiology has been met with new and unexpected limitations. Historically, neuropharmacology research has used observational and invasive experimental approaches to identify novel therapeutics. Unfortunately, this classic approach suffers from laborious chemical synthesis and in vivo testing which ultimately leads to translational bottlenecks. With the implementation of AI, we are now able to expedite this early testing by modeling how a drug or protein complex may interact with a receptor of interest. By applying powerful, precision-based protein structure prediction tools, we can better tailor therapeutics and minimize undesired outcomes. Though promising, important caveats like predicting chirality of molecules, conformational changes upon binding, and determining downstream signaling elements remain critical roadblocks that functionally limit the efficacy of prediction software. This Perspective article will briefly discuss how AI-powered protein prediction software will impact drug development to transform neuropsychopharmacology research and therapeutics, while also providing insights into the limitations of these digital tools.

## Lay summary

This article explores how artificial intelligence (AI) is changing the way we discover new treatments for brain and mental health disorders. Instead of relying solely on slow and complex lab work, scientists are now using AI to predict how drugs interact with biomolecules. This could speed up the development of safer, more targeted medicines. However, challenges remain, and the article also explains the current limitations of these AI tools in neuropsychiatric research.

## Classical approaches for designing novel biomolecules

In 1865, Crum-Brown and Fraser proposed the idea of structure-activity relationships (SARs) [1]. SARs characterize the relationship between a chemical structure and its predicted biological activity, giving scientists a framework from which to target the efficacy and potency of biological and chemical molecules. By experimentally changing these elements, we can design molecules that leverage or avoid specific therapeutic outcomes (i.e. reduce cytotoxicity or increase potency). Modern applications of this theory integrate mathematical relationships between the chemical molecule and its biological activity, known as quantitative structure-activity relationships (QSARs) [2]. Over time, QSARs evolved into sophisticated machine learning (ML)-based techniques that extract massive data sets, thereby becoming a vital component in drug discovery [3]. These biomolecular quantitative advances continue moving in all directions and have expanded today’s biotechnological landscape. Due to these breakthroughs, we are now able to apply these techniques to study proteins and other biomolecules, changing how we approach neuropsychopharmacology.

Strings of amino acids can be classified into a variety of categories, each of which help define the architectural components or physiological roles of the molecule. The resulting protein provides a complex conformation that can have numerous functional iterations, each of which may interact with other proteins or signaling molecules [4]. The precise nature of these interactions is largely shaped by protein folding and unfolding, thereby representing a critical mechanism for biomolecular activity in live organisms [5]. However, while protein folding itself is understood to be essential to cellular signaling, our ability to model, predict, and ultimately develop novel compounds to mimic these processes has been significantly more difficult. From a structural perspective, the use of artificial intelligence (AI) has allowed us to make some headway to readily develop 3D protein structures with high accuracy [6, 7]. This work has evolved since the first attempts to visualize protein molecules with plasticine models. While plasticine modeling enabled the visualization for folding, its utility was limited to proteins with simple structures. X-ray crystallography and electron microscopy catapulted that visualization to in-depth realistic patterns that were obtained with the use of computation [8]. Though useful for understanding an isolated protein, it still leaves unresolved how the protein may change in response to interactions with other bioactive compounds. This is why it took decades to generate protein models in the last century; there was no way to visualize how amino acids would bind and fold given the numerous tertiary structures or shapes they could assume [9].

## The advent of biomolecule prediction software

AI development has refined machine learning (ML) and deep learning capabilities providing powerful algorithms that are changing science. ML, a subfield of AI, works by recognizing patterns in an extensive data set, allowing the system to learn and improve [10, 11]. Although it requires the programmer to set a defined task, it does not need explicit step-by-step instructions. Instead, ML modules allow systems to learn and interpret bioinformatics data to improve past models [12]. This process allows the program to train on existing data and significantly reduce errors. Deep learning (DL), a sublayer of ML, employs neural networks (layers of structure and functional analysis inspired by animal brains) to process unstructured data (i.e. incomplete or diverse information) in various forms while performing complex tasks [13]. Optimized DL, which adjusts parameters to filter out unnecessary information, enables protein structure design that may or may not have a predetermined shape or function. This allows the program to consider diverse characteristics like the physical energy forces that play a crucial role on protein conformation.

DL has resulted in the development of an artificial intelligence program called AlphaFold, which aims to address protein folding and predict 3D protein structures [14, 15]. There are two previous versions of AlphaFold: AlphaFold 1 and AlphaFold 2. AlphaFold 1 was released in 2018, using neural networks to predict protein structures, while AlphaFold 2, released in 2021, switched its ML algorithm to continuously refine locations of nearby amino acids for every new input, and could predict structures with atomic level accuracy [16, 17]. Although Alpha Fold 2 could model protein structures and their interactions with higher accuracy, it lacked the ability to predict complexes involving a broader range of biomolecules [18–20]. The latest version of the software, AlphaFold 3 (AF), released in 2024 can infer and simulate protein-protein interactions, protein-ligand interactions, small molecules, nucleic acids, ions and modified residues [21]. AF works by being fed lists of molecules to learn and estimate confidence ranges, values within which the model approximates the actual structure. It then compares the sequence entered by the user to known protein structures in databases (i.e. Protein Data Bank) to align molecular data to existing templates. Once AF understands the biomolecular framework being used, it activates its unique diffusion network. AF’s diffusion network generates possible conformations and provides highly accurate molecular structures by applying chemical and physical limits, such as molecular forces and bond lengths [22]. It uses the templates to guide the folding process of new proteins thereby estimating how sequences, and then complexes, could look once assembled. AF goes further by not relying on homology modeling (how related proteins would look) to visualize biomolecular complexes as its programming identifies patterns, infers biochemical properties and predicts 1D, 2D and 3D structures from primary biomolecular sequences. It bypasses homology altogether by successfully rendering graphical representations without using template structures while predicting previously unknown protein folds [23].

**Fig. 1:**
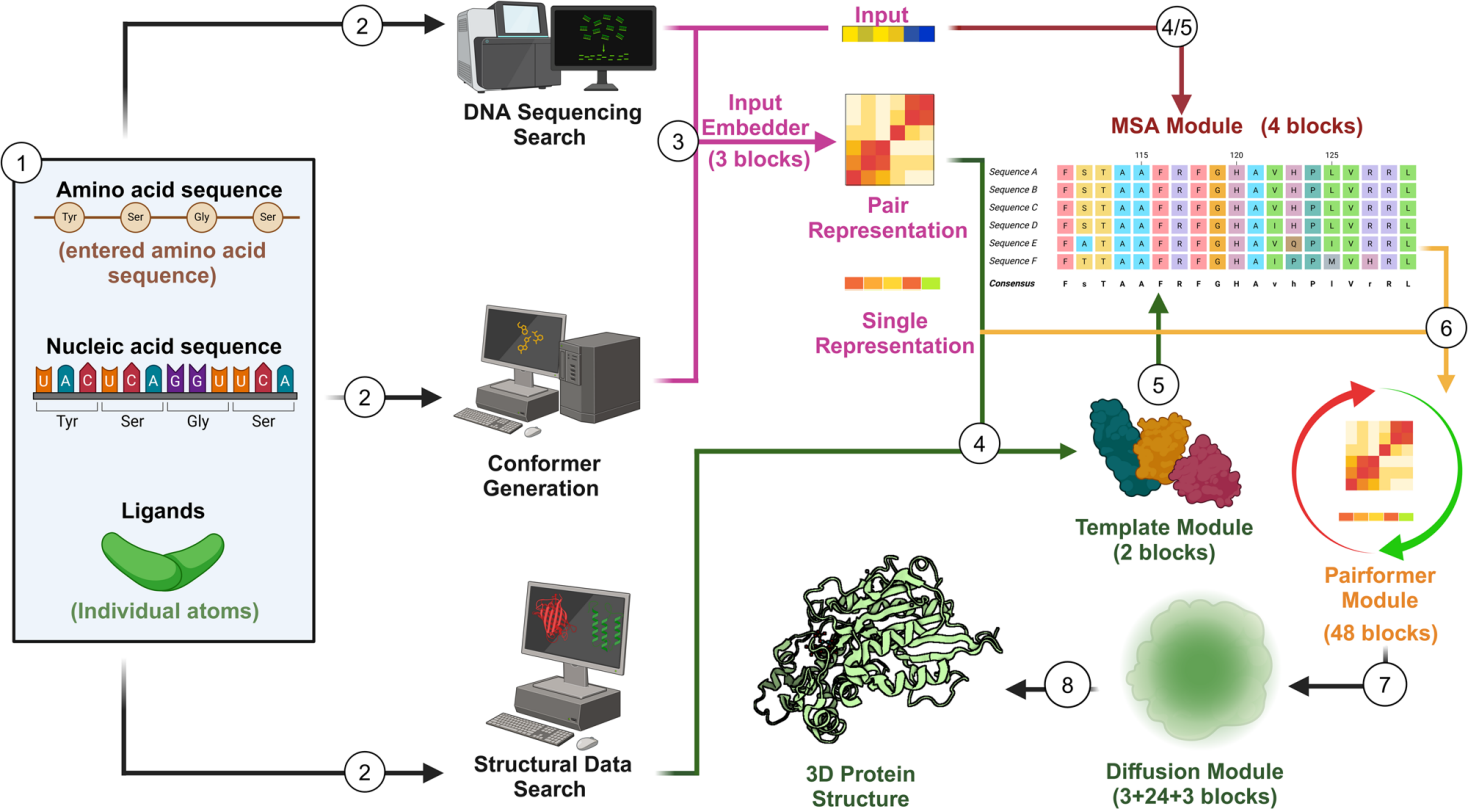
Schematic of AlphaFold 3 mechanism. AlphaFold 3 can predict the structure of proteins, DNA/RNA molecules, and ligands. 1) Once a sequence has been entered, 2) the software concurrently engages with multiple databases to assess potential genetic sequences, conformational prototypes, and structural configurations. 3) The input embedder then uses the sequencing and conformer information to encode and generate a composite that results in a single and pair representation. 4) The template module then integrates known structures obtained from the structural data search into the pair representation. 4/5) In tandem, the Multiple Sequence Alignment (MSA) Module incorporates the sequencing, pair representation, and template models to iteratively build novel base templates. 6) From here, the pairformer module uses MSA information to test different interacting elements, refining the predicted molecule interactions and repeatedly updating the pair and single representations. 7) Finally, the diffusion module applies and removes noise into the algorithm to improve local stereochemistry and global structures.

Protein prediction tools in the past were not built to analyze potential scenarios where physical and stereochemical forces interact [24]. Acknowledging physical and stereochemical tendency is essential when morphing complexes as it can completely change conformations or even compatibility [7]. AF approaches this difficulty by having a DL-based structure prediction architecture that has been trained on biomolecular structures to predict its interactions (Fig. 1). AF uses imputed biomolecules in mathematical form to embed descriptive information as there are diverging structures between amino acids and nucleic acids (proteins and DNA/RNA structures, respectively) [25, 26]. AF also maintains realistic bond geometries, minimizes steric clashes, and accounts for physical energy distribution to achieve fidelity in atomic structures [27]. These principles inform it to anticipate constraints in physics that can destabilize the spatial arrangement of atoms that form molecular structures (stereochemical consistency). Further, it prevents impossible configurations to an extent drawing from the same stereochemical principles it has learned and trained on. With these advances, AF predictions of how complex folding would occur are unparalleled as it takes potential manipulations under consideration [28, 29].

Another feature of AF is that it uses biologically driven principles to guide and identify key model characteristics. First, AF screens multiple sequence alignments (MSA) identifying conserved residues (amino acids) across species [30]. This step aids in the process of predicting structurally and functionally important regions. When evolutionarily co-varying positions in these sequences are identified, they can point to potential physical interactions that guide folding and binding predictions. Complexes that function together often evolve together, but this can happen in a myriad of ways [31]. Identifying points of convergent evolution for how complexes evolve allows us to highlight correlated mutations. These mutations can affect how biomolecules interact with neural cells and receptors at interfaces where differences in complex binding can have unintended consequences. In testing how biomolecules achieve conformation states, modular adaptations to the structure and functional regions helps AF predict new folding patterns even when direct homologs are unknown. In sum, AF continues to build on earlier and similar iterations but uniquely incorporates a more flexible design that allows for exciting new opportunities for drug discovery.

## Current applications of biomolecule prediction software

**Fig. 2:**
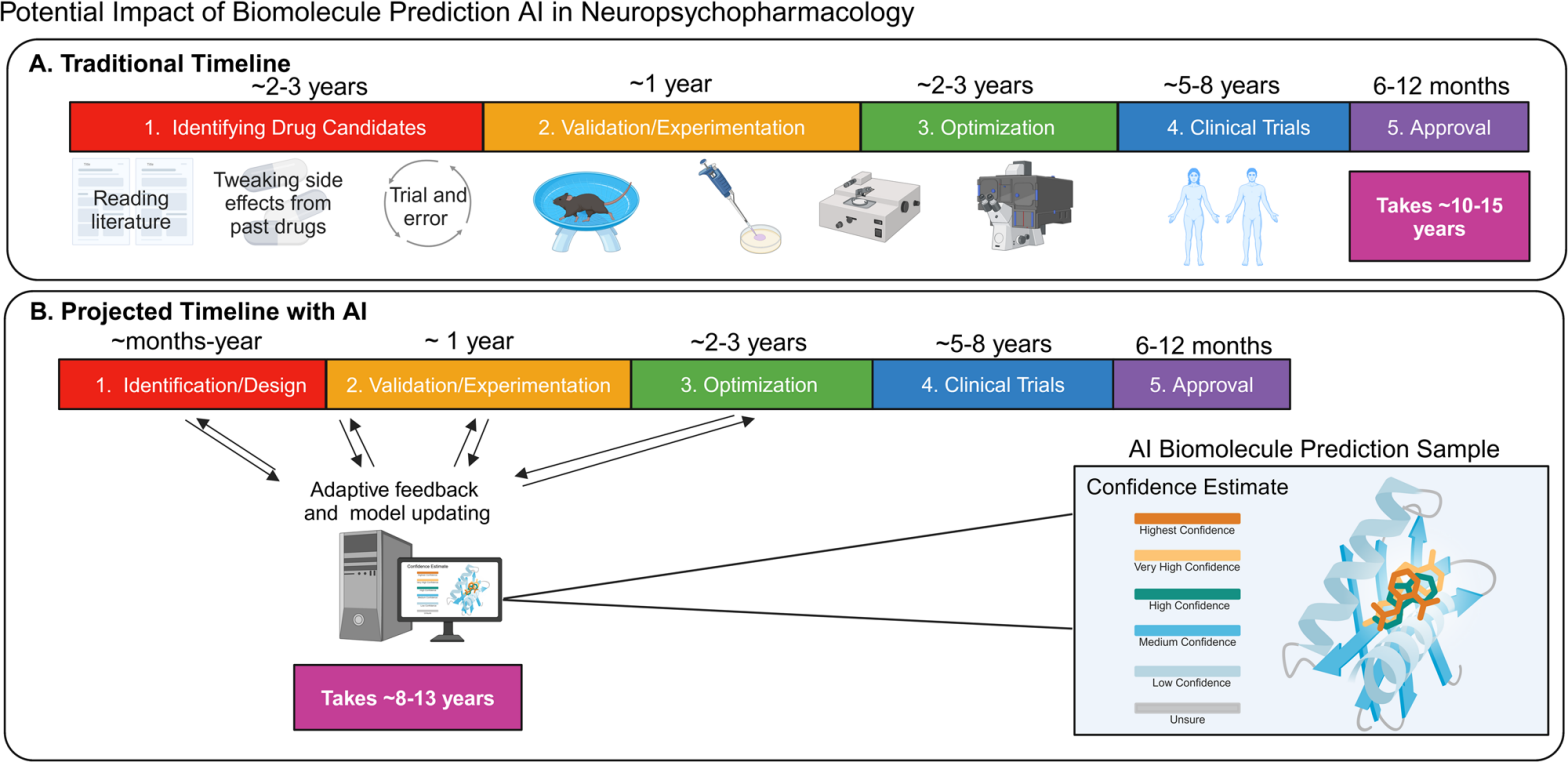
Comparison of traditional versus AI assisted drug development pipelines. **A** Traditional timeline for creating new drugs. A1–5) Partitioned timeline showing major categories of drug development (vivid colors). Subcategories describing unique elements are below major categories. Overall timeline from drug candidate identification to approval is ~10–15 years. **B** Projected timeline for creating new drugs with AI assistance. B1–5) Same as A1–5, but with notably shorter durations driven by continued AI optimization at each step. Generative AI models based on validation and clinical evidence can enhance the trajectory to approval by ~3 years.

Neuropsychiatric disease research benefits from AF by having the ability to improve target modeling and drug development. More specifically, AF can shorten time windows to de-risk biomolecules for toxicity and adverse events through profiling drug candidates (Fig. 2), assist in the isolation and characterization of novel g protein-coupled receptors (GPCRs), and, when empowered in the near future, potentially anticipate unexpected problems during biomolecule complex folding (i.e. misfolding) [32–34]. Currently, AF models outperform traditional modeling (i.e. homology) tools ~70% of the time, performing particularly well on “hard targets” (proteins with no known homologs). Evidence for its advantage in this sphere has been observed at Critical Assessment of Protein Structure Prediction (CASP) competitions. Here, prediction software candidates are evaluated on multiple readouts and categories, such as the global distance test (GDT). GDT assesses how close the predicted protein structure is to an experimentally determined structure, thus providing a ground truth comparison. Over time, AF has improved its score, with a recent 2020 competition scoring on par with experimentally determined structures [35].

One real world example of AF in action involves the discovery of a ligand for the trace amine–associated receptor 1 (TAAR1), a G protein–coupled receptor that has an unknown structure and is a promising target for treating neuropsychiatric disorders ranging from schizophrenia to substance use disorder [32]. TAAR1 receptors are found both in the central nervous system and in the periphery [36, 37]. TAAR1 modulates monoaminergic systems, particularly dopamine, serotonin, and norepinephrine. Unlike conventional antipsychotics that block D2 dopamine receptors, TAAR1 activation modulates dopaminergic tone indirectly, reducing hyperdopaminergia (persistent increase in central dopaminergic transmission) without causing motor side effects like tardive dyskinesia (a common side effect of antipsychotics). In the periphery, emerging evidence suggests that TAAR1 activation is involved in insulin secretion (pancreas) [38], energy homeostasis, and body weight regulation while regulating neural circuits [39]. This raises interest in TAAR1 as a potential target for metabolic disorders like type 2 diabetes and obesity. Considering its wide utility, there is a strong interest in developing compounds to modulate this receptor. Unfortunately, drug discovery for GPCRs like TAAR1 has been hampered by the lack of high-resolution structural data. While this is typically a major obstacle for traditional, homology-based approaches, AF was able generate a high-confidence structural model of TAAR1, enabling researchers to speed up structure-based virtual screening. Researchers computationally docked over 16 million fragment-like molecules from the ZINC database into the estimated binding site of TAAR1. The AF-derived structure identified 6.8 million compounds capable of docking at the binding site, from which 30 top-ranked candidates were selected for experimental testing. The most potent and selective compound from the AF screen, referred to as compound 65, displayed good selectivity against other aminergic receptors and favorable pharmacokinetic properties. Compound 65 was further validated using in vivo in mouse models. They found that compound 65 regulated body temperature and produced sensorimotor gating behavior (whole body flinch), which is an animal model proxy for antipsychotic-like effects. These effects were absent in TAAR1 knockout mice, confirming that the observed pharmacology was mediated through TAAR1 activation. Throughout this process, AF was tested against a homology-based model to benchmark drug discovery efficacy. They found that the AF-derived model delivered a 60% hit rate, nearly three-fold higher compared to the 22% hit rate of the homology model. Cases like these highlight the strengths of this new generation of prediction software, particularly as it relates to the early phases of drug discovery.

## Current limitations of biomolecule prediction software

It is clear that biomolecule prediction software represents an important leap in biomedical AI technology [40]. Accurately predicting structures and examining their interaction in a unified framework will reduce time frames to search for or design new drug candidates. However, these models still have several critical areas in which continued development is necessary, such as their preponderance to create spurious structural orders (also called hallucinations [41]). These digital hallucinations are predicted structural features that may appear well-formed and confident in the model but do not actually exist in the real, native structure of the protein—often due to limitations or artifacts in the model’s training or input data. Troubleshooting these and similar problems (e.g., incomplete/disorganized bioinformatic data) will help alleviate these disruptions in future models as human intervention exerts guidance in aiding accurate simulations.

AF excels at predicting interaction between structures (i.e., between a ligand and a receptor), but not at quantifying affinity or potency (i.e., the effect of that interaction). To assess that, additional modeling tools or experimental data are required. As such, AF is best viewed as a high-resolution input for further computational or wet-lab work rather than a standalone solution for predicting drug efficacy. Relatedly, AF also struggles to incorporate extracellular space conditions that may impact molecular interactions. The accuracy drops when handling chirality and chiral molecules (binding and arrangement of atoms) as the algorithm attempts to predict molecular interactions [21, 23]. It integrates some basic chemistry, but as of now does not extrapolate essential spatial distributions to assemble molecules that can be easily or realistically synthesized in the lab [14, 42, 43]. As a result, it often omits crucial components like heme groups, misrepresents metal coordination sites, and cannot account for covalent drug binding or enzymatic active site geometry.

Finally, AF exhibits limitations when modeling intrinsically disordered regions, non-specific protein interactions, and proteins with significant conformational flexibility. Despite attempts to incorporate AI techniques from image generation, such as diffusion models, AF can still produce structurally implausible features like atom overlaps or steric clashes [44]. It also occasionally omits key structural elements, necessitating expert human correction. For effective drug design, AF would benefit from better integration of principles from medicinal chemistry, structural biology, and physics to more accurately reflect real-world molecular forces and docking behavior. Human oversight remains essential to guide these models and interpret results in a realistic biochemical context. Ultimately, predictions must be validated through experimental assays, and researchers are advised to generate multiple models and compare outputs to increase reliability and identify consistent structural features.

## Discussion

Diagnostically, biomolecule prediction software can provide insights into how genetic mutations impact protein structures and interactions [45]. By understanding how discrete mutations influence protein function, we can better design molecules that account for those functional changes. Still, while AI technology can provide useful suggestions, in vivo testing of molecules remains the gold standard for guiding the selection of drug candidates for clinical trials. Derisking and reducing toxicity still benefits from testing how model organisms respond to biomolecules that are designed to target specific sites for therapeutic effects [46]. However, with the guidance of prediction software, back-end drug development can be significantly improved and expedited. Looking forward, it will be necessary to eliminate the existing disconnect between software developers and software users. Realistic guardrails are essential in AI development and deployment, and critical precautions must be taken to protect the extent to which technology can intervene and execute functions currently handled by humans. This will only be achieved if digital literacy in neuropsychopharmacology research is modernized to implement these types of tools. As an interdisciplinary field, neuropsychopharmacology stands to significantly benefit from optimized novel drug discovery, complex biobehavioral modeling, and standardized yet precision-based treatments. The adoption of AI into neuropsychiatric disease treatment will be a difficult, but important, conversation amongst both those administering and receiving treatments. However, we believe that an open discussion for how and when to incorporate innovative AI technology represents an exciting step forward in precision medicine.

### Citation diversity statement

The authors have attested that they made efforts to be mindful of diversity in selecting the citations used in this article.
